# *Centrorhynchus* spp. (Acanthocephala) in South America: new anuran record and checklist of vertebrate hosts

**DOI:** 10.1590/S1984-29612024024

**Published:** 2024-05-31

**Authors:** Róger Jean Oliveira, Carolina Silveira Mascarenhas, Gertrud Müller

**Affiliations:** 1 Laboratório de Parasitologia de Animais Silvestres, Departamento de Microbiologia e Parasitologia, Instituto de Biologia, Universidade Federal de Pelotas – UFPel, Pelotas, RS, Brasil; 2 Instituto Federal Sul-Rio-Grandense – IFSUL, Pelotas, RS, Brasil

**Keywords:** Bullfrog, cystacanth, paratenic host, definitive host, invader, infection rates, Rã-touro, cistacanto, hospedeiro paratênico, hospedeiro definitivo, invasora, índices de infecção

## Abstract

The aim of this study was to record *Centrorhynchus* sp. associated with the exotic species *Aquarana catesbeiana* (bullfrog) in southern Brazil and to present a checklist of vertebrate hosts in South America. Twenty-nine adults and juveniles of *A. catesbeiana* were collected in Capão do Leão, state of Rio Grande do Sul, Brazil, between October 2019 and December 2020. We found 275 specimens of *Centrorhynchus* sp. cystacanths in the stomach musculature and coelomic cavity of 55.1% of hosts (16). There was no significant differences in the prevalence and mean intensity of infection with cystacanths when compared males and females of *A. catesbeiana*. The prevalence was significantly higher in adults than in juveniles. The checklist presents 106 species of vertebrate hosts and 14 taxa of *Centrorhynchus* recorded in nine South American countries. Avian were the main definitive hosts of *Centrorhynchus* spp. and snakes Dipsadidae, anurans Hylidae and Leptodactylidae the main paratenic hosts in South America. This is the first record of *Centrorhynchus* cystacanths in *A.* *catesbeiana* in the South America. The study provides tools to help understand the parasitic relationships between species of *Centrorhynchus* and *A. catesbeiana* and other hosts in areas where bullfrog have been introduced.

## Resumo

O objetivo deste estudo foi registrar *Centrorhynchus* sp. associado à exótica *A. catesbeiana,* no extremo sul do Brasil, e apresentar uma checklist de hospedeiros vertebrados na América do Sul. Vinte e nove adultos e juvenis de *A. catesbeiana* foram coletados em Capão do Leão, estado do Rio Grande do Sul, Brasil, entre outubro de 2019 e dezembro de 2020. Foram encontrados 275 cistacantos de *Centrorhynchus* sp. na musculatura do estômago e na cavidade celômica de 55,1% dos hospedeiros (16). Não houve diferença significativa na prevalência e intensidade média de infecção de cistacantos entre machos e fêmeas de *A. catesbeiana*. A prevalência foi significativamente maior em anuros adultos que em juvenis. O checklist apresentou 106 espécies de hospedeiros vertebrados e 14 taxa de *Centrorhynchus* registrados em nove países da América do Sul. Aves são os principais hospedeiros definitivos de *Centrorhynchus* spp., e serpentes Dipsadidae, anuros Hylidae e Leptodactylidae principais hospedeiros paratênicos no continente sul-americano. Este é o primeiro registro de cistacantos de *Centrorhynchus* em *A.* *catesbeiana* na América do Sul. O estudo fornece ferramentas para auxiliar na compreensão das interações parasitárias entre espécies de *Centrorhynchus* e *A. catesbeiana* e outros hospedeiros em áreas onde a rã-touro foi introduzida.

## Introduction

*Centrorhynchus* (Lühe, 1911) (Palaeacanthocephala: Centrorhynchidae) comprises around 100 species, with records from various regions worldwide (Amin, 2013; Smales et al., 2018). *Centrorhynchus* species parasitize the intestines of birds and mammals, which are the definitive hosts (Lunaschi & Drago, 2010). Transmission of these acanthocephalans occurs through the trophic web, since the infective forms (cysthacanths) develop in arthropods (intermediate hosts). Amphibians and reptiles can occasionally participate in the life cycle as paratenic hosts of cystacanths, serving as a trophic bridge between intermediate and definitive hosts (Petrochenko, 1971; Amato et al., 2003).

Bullfrog *Aquarana catesbeiana* (Shaw, 1802) (Anura: Ranidae) due its large size in adulthood native to the northeastern region of the United States and Canada and has been introduced to several countries as a species of commercial interest (Both et al., 2011; Maneyro & Carreira, 2012). In Brazil, this species was introduced in the 1930s and can be found mainly in the southern and southeastern regions (Silva, 2016). Occurrences of cystacanths of *Centrorhynchus* in *A. catesbeiana* have been recorded in Texas and North Carolina in the United States (Brandt, 1936; Hollis, 1972) but there are no records for bullfrogs in South America.

The lack of knowledge about the diversity of helminths associated with the exotic *A. catesbeiana* in Brazil, highlights the importance of helminthological research, since the introduction of host species and parasites can cause imbalances in native ecosystems (Maneyro & Carreira, 2012). The checklist provides a basis for studies on the interactions between *Centrorhynchus* species and their different hosts and life cycle. The aim of the present study was to record cysthacanths parasitizing *A. catesbeiana* in southern Brazil, and to provide a checklist of vertebrate hosts in South America.

## Material and Methods

### Host sampling, parasite collection and identification

Twenty-nine specimens of *A. catesbeiana* (9 males, 10 females and 10 juveniles of undetermined gender) were collected from artificial ponds located in Ichthyology Laboratory of the Department of Animal Science, Universidade Federal de Pelotas (UFPel), in the municipality of Capão do Leão (31°48'13.1"S 52°25'00.8"W), state of Rio Grande do Sul (RS), Brazil, between October 2019 and December 2020. These hosts were collected using a fishing rod with a hook and bait and were euthanized in accordance with Resolution No. 1000 of the Federal Council of Veterinary Medicine (Brasil, 2012).

The bullfrog were packed in individual plastic bags and transported to the Laboratory. The hosts were refrigerated, measured for the total snout-vent length (SVL), weighted and necropsied. Five were necropsied and examined after refrigeration and the others (which had been frozen), after thawing. We examined host gender during necropsies, observing the gonads and considering external sexual dimorphism. Hosts stage of maturation (juveniles or adults) were defined according to Quiroga et al. (2015).

We examined separetely all internal organs, and the cystacanths collected were remove from cysts and transferred to cold tap water to extrovert the proboscis. Subsequently fixed in AFA solution (92% 70°GL ethanol + 5% formalin + 3% acetic acid), preserved in 70ºGL ethanol and stained with hydrochloric carmine, clarified with creosote and mounted in Canada balsam (Amato & Amato, 2010).

The measurements of six specimens are in micrometers (unless when indicated) and are presented as range of values and mean in parenthesis. Photomicrographs were prepared using an Olympus BX 41 microscope with an attached camera and figures were created using Adobe Photoshop CS5. Representative specimens were deposited in the Helminth Collection of the Laboratory of Wild Animal Parasitology (LAPASIL) of the Universidade Federal de Pelotas (CHLAPASIL 953-958) and in the Helminthological Collection of the Oswaldo Cruz Institute (CHIOC 39614-39615, 39948).

Prevalence (P%), mean intensity of infection (MII) and mean abundance (MA) were estimated as described by Bush et al. (1997). The influence of host gender (males and females) and stage of maturation (juvenile and adults) in prevalence of cysthacanths were tested using Fisher's exact test (p ≤ 0.05) while the effect of those host factors in MII was analyzed by *t*-test (p≤ 0.05) using the Quantitative Parasitology software (QPweb) (Reiczigel et al., 2019).

### Checklist elaboration

The checklist used information compiled from Google Scholar, Pubmed, and Scielo. The databases used words such as: “*Centrorhynchus* and/or Acanthocephalans in South America”; “*Centrorhynchus* and/or Acanthocephalans in vertebrates of Brazil” (Argentina, Uruguay, Chile, Paraguay, Bolivia, Peru, Ecuador, Colombia, Venezuela, Guyana e Suriname); checklist of the *Centrorhynchus* and/or Acanthocephalans in South America”.

The vertebrate hosts were organized as definitive and paratenic hosts, and presented in alphabetical order within their respective families along with the *Centrorhynchus* taxon recorded. The classification and systematization of the hosts followed Costa et al. (2021), Pacheco et al. (2021), Abreu et al. (2021), IUCN (2022), Segalla et al. (2021), Frost (2023), Remsen et al. (2023) and Uetz et al. (2023).

Each record includes the name of the *Centrorhynchus* species, the authority and year. Host species are recorded in alphabetical order and, where possible, followed by the locality of the record in parenthesis, site of infection and life stage. The references are indicated in chronological order. The abbreviations for all localities cited in the work are:

**Argentina -** Pirané (PIR), Formosa Province (FOP), Villa la Angostura (VLA), La Rinconada (LAR), Bariloche City (BAC), Villa Mascardi (VIM), Mburucuyá (MBU), Ituzaingó (ITU), Corrientes (COR) and Chaco Province (CHP).
**Brazil -** Minas Gerais (MG), Rio Grande do Sul (RS), Mato Grosso (MT), Paraíba (PB), Paraná (PR), Rio de Janeiro (RJ), São Paulo (SP), Pernambuco (PE), Amazonas (AM), Pará (PA) and Ceará (CE).
**Chile -** Valparaíso (VAL), Biobío region (BIO), Chillán, Ñuble region (CNR), Altos de Cantillana Natural Reserve (CAN), Isla Teja (IST) and Island of Chiloé (ISC).
**Colombia -** Meta (MET), Anchicaya Valle (ANV), Choco Province (CHP).
**Paraguay -** Itaipu (ITA), Santa Maria (STM), Arroyo Aguapey (ARA), Alto Paraguay Province (APP), Concepcion Province (COP), Presidente Hayes Province (PHP), Cordillera Province (CPR), Paraguari Province (PAP), Itapua Province (ITP), Transchaco (TR), Cerrito (CER) and Aquidaban (AQU).
**Peru -** San Martin (SMA) and Chulucanas (CHU).
**Uruguay -** Montevideu (MVD).
**Venezuela** **-** Venezuela (VEN).

## Results

### *Centrorhynchus* cystacanths in *Aquarana catesbeiana*

Two hundred and seventy-five cystacanths of *Centrorhynchus* sp. were found in the stomach musculature (4 host) and celomic cavity (16 host) of the anurans. The overall prevalence was 55.1%, while the mean intensity and mean abundance of infection were 17.1 helminths/host and 9.4, respectively. There was no significant difference in the prevalence and mean intensity of cystacanth infection between males and females of *A. catesbeiana* (Table 1). However, the prevalence of helminths in adult bullfrogs was significantly higher than in juvenile (Table 2).

**Table 1:** Prevalence (P%), mean intensity of infection (MII), mean abundance (MA) and intensity of infection (INI) of *Centrorhynchus* sp. cystacanths parasitizing females and males of *Aquarana catesbeiana* (Anura: Ranidae) in southern Brazil.

**Infection parameters**	**Females (n=10)**	**Males (n=9)**
P (%)	60.0	100
MII	13.6	21.2
MA	8.2	21.2
INI	1-32	1-69

n = number of specimens analyzed.

**Table 2:** Prevalence (P%), mean intensity of infection (MII), mean abundance (MA) and intensity of infection (INI) of *Centrorhynchus* sp. cystacanths parasitizing adults and juveniles of *Aquarana catesbeiana* (Anura: Ranidae) in southern Brazil.

**Infection parameters**	**Adults (n=19)**	**Juveniles (n=10)**
P(%)	78.9 *	10 *
MII	18.2	2
MA	14.3	0.2
INI	1-69	2

n = number of specimens analyzed;

* p = 0.001 using Fisher's exact test.

A taxonomic summary of results is provided below.

Family Centrorhynchidae Van Cleave, 1916

Genus *Centrorhynchus* Lühe, 1911

*Centrorhynchus* sp.

Host: *Aquarana catesbeiana* (Shaw, 1802)

Developmental stage: Cystacanth

Site of infection: Stomach musculature and celomic cavity.

Prevalence, mean abundance and mean intensity of infection: 55.1% (16/29), 9.4 and 17.1 helminths/host, respectively.

Locality: Capão do Leão, state of Rio Grande do Sul, Brazil.

Specimens deposited: 953-958 (CHLAPASIL); 39614-39615, 39948 (CHIOC).

**Figure 1:**
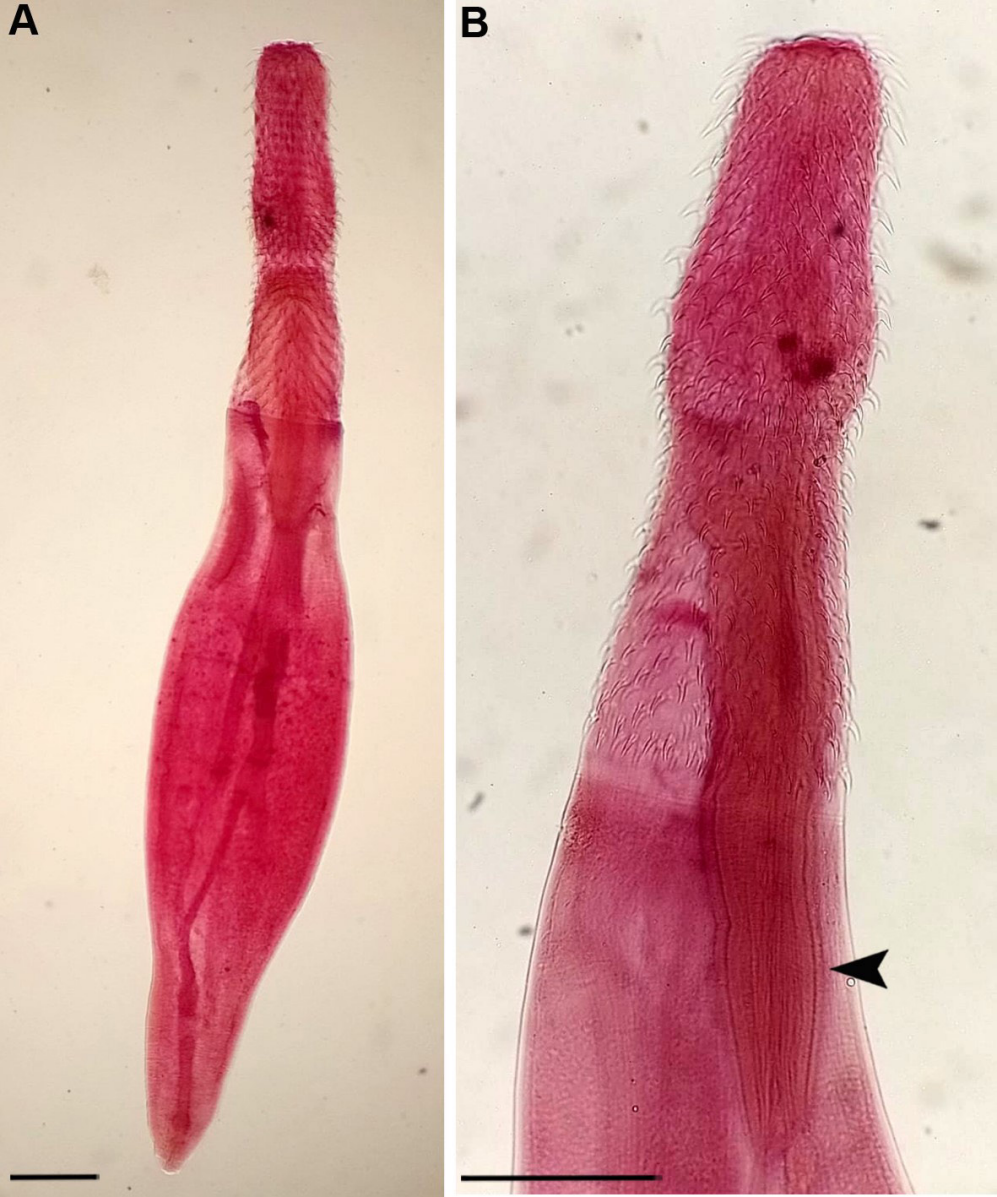
Cystacanths of *Centrorhynchus* sp. in *Aquarana catesbeiana* (Anura: Ranidae) in southern Brazil. **A -** General view (scale-bar: 300); **B -** Detail of proboscis, arrow = proboscis receptacle (Scale-bar: 300).

Description (based in six specimens): Body elongated, filiform. Proboscis divided into three portions, first quadrangular, second inflated; third elongated and wider; and with a constriction at the insertion of the double-walled proboscis receptacle. Proboscis with 28 - 30 longitudinal rows of hooks, each row consisting of 21 -23 hooks. Body length 1.99 4.34 (3.28 millimeters); wider portion of body 460-750 (581). Cylindrical proboscis, 790-1,170 (1,021) in length; proboscis width: at anterior end 150 - 230 (206); at inflated portion 200 - 310 (258); at level of receptacle insertion 170 - 260 (225); and at base 270 - 370 (313). Length of apical hooks 43 - 60 (50); root length of apical hooks 33 - 45 (38). Length of basal spines 33- 43 (36). Proboscis receptacle double-walled 620 - 1050 (782) in length, and width 200 - 250 (225). Length of lemniscus 420 640 (530) (only three specimens measured) (Figure 1).

### *Centrorhynchus* species in South America

We found 14 taxa of *Centrorhynchus* and 106 species of vertebrate hosts recorded in nine South American countries. The first records date back to the beginning of the 20^th^ century, with a greater number of reports published from the 2000s (see checklist).

**Figure 2:**
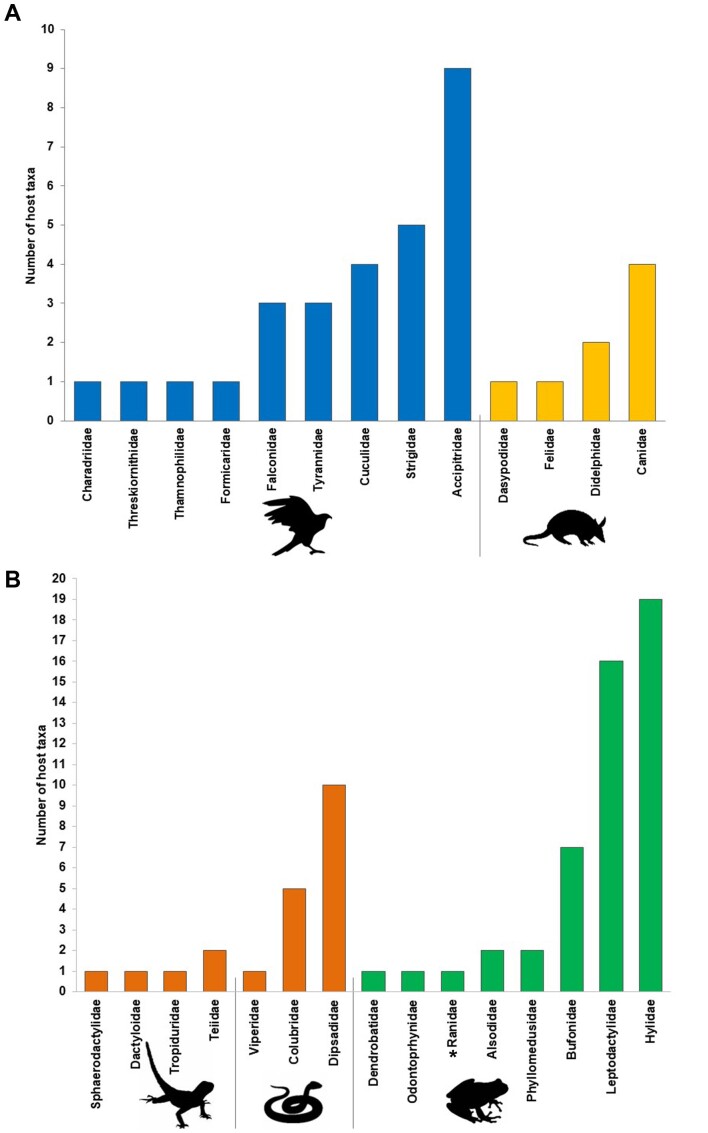
**A -** Number of species reported as definitive hosts of *Centrorhynchus* spp. per host family. General Falconidae shape represent the avian and general Dasypodidae shape represent Mammal families; **B -** Number of species reported as paratenic hosts of *Centrorhynchus* spp. per host family in South America. General Tropiduridae shape represent the lizard families, snake shape represent snake families and Hylidae shape represent anuran families. Ranidae - record of the present study.

**Figure 3:**
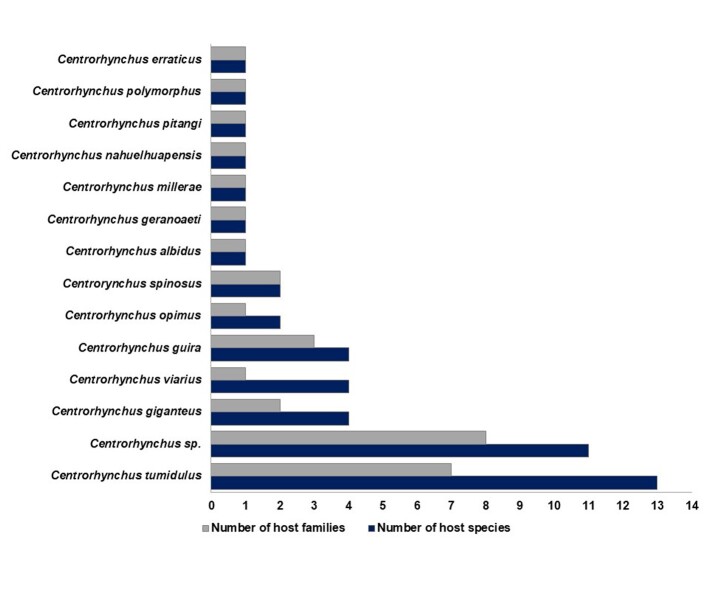
Species of *Centorhynchus* reported in definitive hosts by number of host families and number of host species.

We list 28 species of birds in association with at least one *Centrorhynchus* species. Accipitridae and Strigidae have been reported as hosts for five taxa (Table 3), while Accipitridae (nine taxa) is the group with the greatest diversity of birds recorded in association with *Centrorhynchus* spp. (Figure 2). Mammals (eight species) have were recorded as hosts for five *Centrorhynchus* taxa (Table 3). *Centrorhynchus* *tumidulus* (Rudolphi, 1819) was found in different groups of definitive hosts (6 birds families and 1 mammal family) predominating in number of host species in South American countries (Figure 3 and Table 3).

**Table 3:** Records of bird and mammal hosts of *Centrorhynchus* (Lühe, 1911) species in South America.

**Hosts**	**Species of *Centrorhynchus***
**Aves**	
**Accipitridae**	
*Geranoaetus melanoleucus* (Vieillot, 1819)	*Centrorhynchus geranoaeti* (Smales, 2013)
*Geranoaetus polyosoma* (Quoy & Gaimard, 1824)	*Centrorhynchus* sp.
*Heterospizias meridionalis* (Latham, 1790)	*Centrorhynchus giganteus* (Travassos, 1919)
	*Centrorhynchus tumidulus* (Rudolphi, 1819)
	*Centrorhynchus viarius* (Smales, 2013)
*Leptodon cayanensis* (Latham, 1790)	*Centrorhynchus giganteus* (Travassos, 1919)
*Leucopternis princeps* (Sclater, 1865)	*Centrorhynchus* sp.
*Parabuteo unicinctus* (Temminck, 1824)	*Centrorhynchus* sp.
	*Centrorhynchus viarius* (Smales, 2013)
*Pseudastur albicollis* (Latham, 1790)	*Centrorhynchus tumidulus* (Rudolphi, 1919)
*Rupornis magnirostris* (Gmelin, 1788)	*Centrorhynchus tumidulus* (Rudolphi, 1919)
	*Centrorhynchus giganteus* (Travassos, 1919)
	*Centrorhynchus viarius* (Smales, 2013)
*Urubitinga urubitinga* (Gmelin, 1788)	*Centrorhynchus viarius* (Smales, 2013)
**Charadriidae**	
*Vanellus chilensis* (Molina, 1782)	*Centrorhynchus* sp.
**Cuculidae**	
*Coccyzus melacoryphus* (Vieillot, 1817)	*Centrorhynchus tumidulus* (Rudolphi, 1919)
*Crotophaga ani* (Linnaeus, 1758)	*Centrorhynchus guira* (Lunaschi & Drago, 2010) *Centrorhynchus tumidulus* (Rudolphi, 1919)
	*Centrorhynchus* sp.
*Crotophaga major* (Gmelin, 1788)	*Centrorhynchus tumidulus* (Rudolphi, 1919)
*Guira guira* (Gmelin,1788)	*Centrorhynchus tumidulus* (Rudolphi, 1919)
	*Centrorhynchus guira* (Lunaschi & Drago, 2010)
**Falconidae**	
Falconidae gen. sp.	*Centrorhynchus albidus* (Meyer, 1932)
	*Centrorhynchus giganteus* (Travassos, 1919)
*Herpetotheres cachinnans* (Linnaeus, 1758)	*Centrorhynchus* sp.
*Micrastur ruficollis* (Vieillot, 1817)	*Centrorhynchus polymorphus* (Travassos, 1925)
**Formicaridae**	
Formicaridae gen. sp.	*Centrorhynchus tumidulus* (Rudolphi, 1919)
**Strigidae**	
*Asio flammeus* (Pontoppidan, 1763)	*Centrorhynchus tumidulus* (Rudolphi, 1919)
*Athene cunicularia* (Molina, 1782)	*Centrorhynchus* sp.
*Bubo magellanicus* (Lesson,1828)	*Centrorynchus spinosus* (Kaiser, 1893)
*Megascops choliba* (Vieillot, 1817)	*Centrorhynchus tumidulus* (Rudolphi, 1919) *Centrorhynchus millerae* (Smales, 2013)
*Strix rufipes* (King, 1828)	*Centrorhynchus nahuelhuapensis* (Steinauer, Flores & Rauque, 2019)
**Thamnophilidae**	
*Batara cinerea* (Vieillot, 1819)	*Centrorhynchus tumidulus* (Rudolphi, 1919)
**Threskiornithidae**	
*Theristicus caudatus* (Boddaert, 1783)	*Centrorhynchus guira* (Lunaschi & Drago, 2010)
**Tyrannidae**	
*Attila rufus* (Vieillot, 1819)	*Centrorhynchus tumidulus* (Rudolphi, 1819)
*Megarynchus pitangua* (Linnaeus, 1766)	*Centrorhynchus opimus* (Travassos, 1919)
*Pitangus sulphuratus* (Linnaeus, 1766)	*Centrorhynchus opimus* (Travassos, 1919)
	*Centrorhynchus pitangi* (Smales, 2013)
**Mammalia**	
**Canidae**	
*Cerdocyon thous* (Linnaeus, 1766)	*Centrorhynchus* sp.
*Chrysocyon brachyurus* (Illiger, 1815)	*Centrorhynchus* *guira* (Lunaschi & Drago, 2010)
*Lycalopex culpaeus* (Molina, 1782)	*Centrorhynchus spinosus* (Kaiser, 1893)
*Lycalopex gymnocercus* (Fischer, 1814)	*Centrorhynchus* sp.
**Dasypodidae**	
*Dasypus novemcinctus* (Linnaeus, 1758)	*Centrorhynchus* sp.
**Didelphidae**	
Didelphidae gen. sp.	*Centrorhynchus tumidulus* (Rudolphi, 1819)
*Didelphis albiventris* (Lund, 1840)	*Centrorhynchus* sp.
**Felidae**	
*Felis silvestris catus* (Linnaeus, 1758)	*Centrorhynchus erraticus* (Chandler, 1925)

The majority of *Centrorhynchus* species associated with definitive hosts were reported in Paraguay (seven taxa), Brazil (six taxa), Argentina (four taxa), Chile (two taxa), Colombia (two taxa), Uruguay (one taxon) and Venezuela (one taxon). No records of acanthocephalans from this group were found in other South American countries.

Considering the species of *Centrorhynchus* that use vertebrates as paratenic hosts, three taxa of *Centrorhynchus* were recorded in 21 species of reptiles and 49 species of amphibians in seven South American countries. Dipsadidae snakes and anurans belonging to Hylidae and Leptodactylidae were the main groups parasitized by cystacanths (Figure 2, Table 4). Cystacanths of *C. tumidulus* and *Centrorhynchus* sp. have been recorded in Brazil, the latter predominating in the majority of reports in this country, as well as in other South American countries (See checklist below).

**Table 4:** Records of paratenic hosts of *Centrorhynchus* (Lühe, 1911) species in South America.

**Hosts**	**Species of *Centrorhynchus***
**Amphibia**	
**Alsodidae**	
*Eupsophus calcaratus* (Günther, 1881)	*Centrorhynchus* sp.
*Eupsophus roseus* (Duméril & Bibron, 1841)	*Centrorhynchus* sp.
**Bufonidae**	
*Atelopus bomolochus* (Peters, 1973)	*Centrorhynchus* sp.
*Melanophryniscus klappenbachi* (Prigioni & Langone, 2010)	*Centrorhynchus* sp.
*Rhinella crucifer* (Wied- Neuwied, 1821)	*Centrorhynchus* *tumidulus* (Rudolphi, 1919)
*Rhinella dorbignyi* (Duméril & Bibron, 1841)	*Centrorhynchus* sp.
*Rhinella diptycha* (Cope, 1862)	*Centrorhynchus* sp.
*Rhinella granulosus* (Spix, 1824)	*Centrorhynchus* sp.
*Rhinella major* (Müller & Hellmich, 1936)	*Centrorhynchus* sp.
**Dendrobatidae**	
*Oophaga histrionica* (Berthold, 1845)	*Centrorhynchus* sp.
**Hylidae**	
*Boana albomarginata* (Spix, 1824)	*Centrorhynchus* sp.
*Boana albopunctata* (Spix, 1824)	*Centrorhynchus* sp*.*
*Boana pulchella* (Duméril & Bibron, 1841)	*Centrorhynchus* sp.
*Boana raniceps* (Cope, 1862)	*Centrorhynchus* sp.
*Dendropsophus branneri* (Cochran, 1948)	*Centrorhynchus* sp.
*Dendropsophus decipiens* (Lutz, 1925)	*Centrorhynchus* sp.
*Dendropsophus elegans* (Wied-Neuwied, 1824)	*Centrorhynchus* sp.
*Dendropsophus haddadi* (Bastos & Pombal, 1996)	*Centrorhynchus* sp.
*Dendropsophus microcephalus* (Cope, 1886)	*Centrorhynchus* sp.
*Dendropsophus minusculus* (Rivero, 1971)	*Centrorhynchus* sp.
*Dendropsophus minutus* (Peters, 1872)	*Centrorhynchus* sp.
*Dendropsophus nanus* (Boulenger, 1889)	*Centrorhynchu*s sp.
*Dendropsophus sanborni* (Schmidt, 1944)	*Centrorhynchus* sp.
*Scinax auratus* (Wied-Neuwied, 1821)	*Centrorhynchus* sp.
*Scinax nasicus* (Cope, 1862)	*Centrorhynchus* sp.
*Scinax nebulosus* (Spix, 1824)	*Centrorhynchus* sp.
*Scinax x-signatus* (Spix, 1824)	*Centrorhynchus* sp*.*
*Trachycephalus mesophaeus* (Hensel, 1867)	*Centrorhynchus tumidulus* (Rudolphi, 1919)
*Trachycephalus typhonius* (Linnaeus, 1758)	*Centrorhynchus* sp.
**Leptodactylidae**	
*Adenomera diptyx* (Boettger, 1885)	*Centrorhynchus* sp.
*Adenomera marmorata* (Steindachner, 1867)	*Centrorhynchus* sp.
*Leptodactylus bufonius* (Boulenger, 1894)	*Centrorhynchus* sp.
*Leptodactylus elenae* (Heyer, 1978)	*Centrorhynchus* sp.
*Leptodactylus latinasus* (Jiménez de la Espada, 1875)	*Centrorhynchus* sp.
*Leptodactylus latrans* (Steffen, 1815)	*Centrorhynchus tumidulus* (Rudolphi, 1919)
	*Centrorhynchus giganteus* (Travassos, 1919)
*Leptodactylus macrosternum* (Gallardo, 1964)	*Centrorhynchus* sp.
*Leptodactylus mystacinus* (Burmeister, 1861)	*Centrorhynchus* sp.
*Leptodactylus pustulatus* (Peters, 1870)	*Centrorhynchus* sp.
*Leptodactylus vastus* (Lutz, 1930)	*Centorhynchus* sp.
*Physalaemus albonotatus* (Steindachner, 1864)	*Centrorhynchus* sp.
*Physalaemus cuvieri* (Fitzinger, 1826)	*Centrorhynchus* sp.
*Physalaemus nattereri* (Steindachner, 1863)	*Centrorhynchus* sp.
*Physalaemus signifer* (Girard, 1853)	*Centrorhynchus* sp.
*Physalaemus soaresi* (Izecksohn, 1965)	*Centrorhynchus* sp.
*Pseudopaludicola boliviana* (Parker, 1927)	*Centrorhynchus* sp.
**Odontoprhynidae**	
*Proceratophrys renalis* (Miranda-Ribeiro, 1920)	*Centrorhynchus* sp.
**Phyllomedusidae**	
*Phyllomedusa sauvagii* (Boulenger, 1882)	*Centrorhynchus* sp.
*Pithecopus nordestinus* (Caramaschi, 2006)	*Centrorhynchus* sp.
**Ranidae**	
*Aquarana catesbeiana* (Shaw, 1802)	*Centrorhynchus* sp.
**Reptilia**	
**Lacertilia**	
**Dactyloidae**	
*Norops fuscoauratus* (D'Orbigny, 1837 in Duméril & Bibron, 1837)	*Centrorhynchus* sp.
**Sphaerodactylidae**	
*Gonatodes concinnatus* (O’shaughnessy,1881)	*Centrorhynchus* sp.
**Teiidae**	
*Ameiva ameiva* (Linnaeus, 1758)	*Centrorhynchus tumidulus* (Rudolphi, 1919)
*Tupinambis teguixin* (Linnaeus, 1758)	*Centrorhynchus tumidulus* (Rudolphi, 1919)
	*Centrorhynchus* sp.
**Tropiduridae**	
*Tropidurus torquatus* (Wied-Neuwied, 1820)	*Centrorhynchus tumidulus* (Rudolphi, 1919)
**Serpentes**	
**Colubridae**	
*Chironius quadricarinatus* (Boie,1827)	*Centrorhynchus tumidulus* (Rudolphi, 1919)
*Chironius scurrulus* (Wagler in Spix, 1824)	*Centrorhynchus* sp.
*Leptophis ahaetulla* (Linnaeus, 1758)	*Centrorhynchus* sp.
*Palusophis bifossatus* (Raddi, 1820)	*Centrorhynchus tumidulus* (Rudolphi, 1919)
*Spilotes pullatus* (Linnaeus, 1758)	*Centrorhynchus* sp.
**Dipsadidae**	
*Echinanthera undulata* (Wied, 1824)	*Centrorhynchus* sp.
*Erythrolamprus poecilogyrus* (Wied-Neuwied,1824)	*Centrorhynchus* sp.
*Erythrolamprus viridis* (Günther, 1862)	*Centrorhynchus* sp.
*Helicops leopardinus* (Schlegel, 1837)	*Centrorhynchus* sp.
*Imantodes cenchoa* (Linnaeus, 1758)	*Centrorhynchus* sp.
*Imantodes lentiferus* (Cope, 1894)	*Centrorhynchus* sp.
*Lygophis lineatus* (Linnaeus, 1758)	*Centrorhynchus* sp.
*Paraphimophis rusticus* (Cope, 1878)	*Centrorhynchus* sp.
*Philodryas olfersii* (Lichtenstein, 1823)	*Centrorhynchus* sp.
*Pseudablabes patagoniensis* (Girard, 1858)	*Centrorhynchus* sp.
**Viperidae**	
*Bothrops* sp.	*Centrorhynchus tumidulus* (Rudolphi, 1919)

## Checklist of bird and mammal hosts of *Centrorhynchus* species in South America

***Centrorhynchus albidus* Meyer, 1932**

Distribution: Paraguay

Host and locality report: Falconidae gen. sp. (locality not reported)

Site of infection: not reported

Stage: not reported

References: Golvan (1956) in Lunaschi & Drago (2010)

***Centrorhynchus erraticus* Chandler, 1925**

Distribution: Brazil

Host and locality report: *Felis silvestris catus* (MT)

Site of infection: small intestine

Stage: adult

References: Ramos et al. (2013)

***Centrorhynchus geranoaeti* Smales, 2013**

Distribution: Paraguay

Host and locality report: *Geranoaetus melanoleucus* (AQU)

Site of infection: small intestine

Stage: adult and juvenile

References: Smales (2013)

***Centrorhyunchus giganteus* Travassos, 1919**

Distribution: Brazil

Host and locality report: Falconidae gen. sp. (SP), *Heterospizias meridionalis* (MG, MT), *Leptodon cayanensis* (SP) and *Rupornis magnirostris* (MT)

Site of infection: intestine and small intestine

Stage: adult

References: Travassos (1926), Machado (1940) and Pinto & Noronha (1972)

***Centrorhynchus guira* Lunaschi & Drago, 2010**

Distribution: Argentina and Paraguay

Host and locality report: Avian - *Crotophaga ani* (STM)*, Guira guira* (PIR), *Theristicus caudatus* (PIR); mammal - *Chrysocyon brachyurus* (MBU, ITU)

Site of infection: intestine and small intestine

Stage: adult

References: Lunaschi & Drago (2010), González et al. (2013), Smales (2013) and Lunaschi et al. (2015)

***Centrorhynchus millerae* Smales, 2013**

Distribution: Paraguay

Host and locality report: *Megascops choliba* (ARA)

Site of infection: small intestine

Stage: adult

References: Smales (2013)

***Centrorhynchus nahuelhuapensis* Steinauer, Flores & Rauque, 2019**

Distribution: Argentina

Host and locality report: *Strix rufipes* (VLA, LAR, BAC, VIM)

Site of infection: intestine

Stage: adult

References: Steinauer et al. (2019)

***Centrorhynchus opimus* Travassos, 1919**

Distribution: Brazil

Host and locality report: *Megarynchus pitangua* (RJ, PA), *Pitangus sulphuratus* (SP)

Site of infection: intestine

Stage: adult

References: Travassos (1926) and Vicente et al. (1983)

***Centrorhynchus pitangi* Smales, 2013**

Distribution: Paraguay

Host and locality report: *Pitangus sulphuratus* (ARA, COP)

Site of infection: small intestine

Stage: adult and juvenile

References: Smales (2013)

***Centrorhynchus polymorphus* Travassos, 1925**

Distribution: Brazil

Host and locality report: *Micrastur ruficollis* (RJ)

Site of infection: small intestine

Stage: adult

References: Travassos (1926) and Petrochenko (1971)

***Centrorynchus spinosus* Kaiser, 1893**

Distribution**:** Chile

Host and locality report: Avian - *Bubo magellanicus* (locality not reported); mammal - *Lycalopex culpaeus* (CAN)

Site of infection: Small intestine and gastrointestinal tract

Stage: adult and juvenile

References: Grandón-Ojeda et al. (2018) and Oyarzún-Ruiz et al. (2020)

***Centrorhynchus tumidulus* Rudolphi, 1819**

Distribution: Argentina, Brazil, Colombia, Uruguay and Venezuela

Host and locality report: Avian - *Asio flammeus* (RJ), *Attila rufus* (RJ), *Batara cinerea* (RJ), *Coccyzus melacoryphus* (locality not reported), *Crotophaga ani* (RJ, MT, VEN locality not reported), *Crotophaga major* (MT), Formicaridae gen. sp. (RJ), *Guira guira* (MT, MVD, ITA), *Heterospizias meridionalis* (MET), *Megascops choliba* (locality not reported), *Pseudastur albicollis* (locality not reported), *Rupornis magnirostris* (RJ, MT, RS, PB); mammals - Didelphidae gen. sp. (locality not reported)

Site of infection: intestine, large intestine and small intestine

Stage: adult

References: Travassos (1926), Cordero (1933), Machado (1940), Thatcher & Nickol (1972), Boero & Boehringer (1967): in Lunaschi & Drago (2010), Díaz Ungría & Gracia Rodrigo (1960) in Lunaschi & Drago (2010), Melo et al. (2013), Smales (2013) and Lignon et al. (2021)

***Centrorhynchus viarius* Smales, 2013**

Distribution: Paraguay

Host and locality report: *Heterospizias meridionalis* (PHP), *Rupornis magnirostris* (PHP, CER), *Parabuteo unicinctus* (PHP), *Urubitinga urubitinga* (PHP)

Site of infection: small intestine

Stage: adult and juvenile

References: Smales (2013)

***Centrorhynchus* sp.**

Distribution: Argentina, Brazil, Chile, Colombia and Paraguay

Host and locality report: Avian - *Athene cunicularia* (FOP), *Crotophaga ani* (MT), *Cerdocyon thous* (RS), *Geranoaetus polyosoma* (VAL, BIO), *Herpetotheres cachinnans* (PHP)*, Leucopternis princeps* (ANV), *Parabuteo unicinctus* (CNR), *Vanellus chilensis* (PR); mammals - *Dasypus novemcinctus* (RS), *Didelphis albiventris* (RS), *Lycalopex gymnocercus* (RS)

Site of infection: large and small intestine

Stage: adult

References: Machado (1940), Thatcher & Nickol (1972), Müller (2005) in Ruas et al. (2008), Ruas et al. (2008), Gomes et al. (2012), Drago et al. (2015), Silveira & Calegaro-Marques (2016), Grandón-Ojeda et al. (2019) and Oyarzún-Ruiz et al. (2022)

## Checklist of paratenic hosts of *Centrorhynchus* species in South America

***Centrorhynchus giganteus* Travassos, 1919**

Distribution: Brazil

Host and locality report: *Leptodactylus latrans* (RJ)

Site of infection: peritoneum

Stage: cystacanth

References: Travassos (1926)

***Centrorhynchus tumidulus* Rudolphi, 1819**

Distribution: Brazil

Host and locality report: amphibians - *Leptodactylus latrans* (RJ), *Rhinella crucifer* (locality not reported), *Trachycephalus mesophaeus* (RJ); snakes - *Bothrops* sp. (locality not reported), *Chironius quadricarinatus* (RJ), *Palusophis bifossatus* (RJ); lizards - *Ameiva ameiva* (locality not reported), *Tropidurus torquatus* (locality not reported), *Tupinambis teguixin* (locality not reported)

Site of infection: peritoneum

Stage: cystacanth

References: Travassos (1926) and Fabio (1982) in Campião et al. (2014)

***Centrorhynchus* sp.**

Distribution: Argentina, Brazil, Chile, Colombia, Ecuador, Paraguay and Peru

Host and locality report: amphibians - *Adenomera diptyx* (FOP, COR), *Adenomera marmorata* (RJ), ***Aquarana catesbeiana* (RS) (present study),** *Atelopus bomolochus* (CHU), *Boana albomarginata* (PE), *Boana albopunctata* (PR), *Boana pulchella* (RS), *Boana raniceps* (CE), *Dendropsophus branneri* (PE), *Dendropsophus decipiens* (PE), *Dendropsophus elegans* (PE), *Dendropsophus haddadi* (PE), *Dendropsophus microcephalus* (AM), *Dendropsophus minusculus* (CE), *Dendropsophus minutus* (CE, PE), *Dendropsophus nanus* (PAP), *Dendropsophus sanborni* (ITP), *Eupsophus calcaratus* (ISC), *Eupsophus roseus* (IST), *Leptodactylus bufonius* (COR), *Leptodactylus elenae* (ITP), *Leptodactylus latinasus* (COR), *Leptodactylus macrosternum* (COR, APP), *Leptodactylus mystacinus* (RJ), *Leptodactylus pustulatus* (CE), *Leptodactylus vastus* (CE), *Melanophryniscus klappenbachi* (CHP), *Phyllomedusa sauvagii* (COP), *Physalaemus albonotatus* (COR), *Physalaemus cuvieri* (CE, ITP), *Physalaemus nattereri* (COP), *Physalaemus signifer* (RJ), *Physalaemus soaresi* (RJ), *Pithecopus nordestinus* (PE), *Proceratophrys renalis* (CE), *Oophaga histrionica* (CHP), *Pseudopaludicola boliviana* (COR), *Rhinella dorbignyi* (APP, RS, COR), *Rhinella diptycha* (CE), *Rhinella granulosa* (APP), *Rhinella major* (PA), *Scinax auratus* (PE)*, Scinax nasicus* (PHP, COR), *Scinax nebulosus* (AM), *Scinax x-signatus* (PE), *Trachycephalus typhonius* (SP); snakes - *Chironius scurrulus* (SMA), *Echinanthera undulata* (SP)*, Erythrolamprus poecilogyrus* (PHP)*, Erythrolamprus viridis* (CE), *Helicops leopardinus* (COP), *Imantodes cenchoa* (locality not reported), *Imantodes lentiferus* (locality not reported), *Leptophis ahaetulla* (COP, COR), *Lygophis lineatus* (CPR), *Paraphimophis rusticus* (locality not reported), *Philodryas olfersii* (RS), *Pseudablabes patagoniensis* (PAP), *Spilotes pullatus* (SMA); lizards - *Gonatodes concinnatus* (locality not reported), *Norops fuscoauratus* (CE) and *Tupinambis teguixin* (APP)

Site of infection: celomic cavity, large and small intestine, body cavity, liver, serous of stomach, stomach, mesentery and gastrointestinal tissues.

Stage: cystacanth

References: Travassos (1926), Fabio (1982) in Campião et al. (2014), Vizcaíno (1993), Torres & Puga (1996), Azevedo-Ramos et al. (1998) in Campião et al. (2014), Puga & Torres (1999), Goldberg & Bursey (2003), Iannacone (2003), Duré et al. (2004), González & Hamann (2006), Hamann et al. (2006a, b), Schaefer et al. (2006); Smales (2007a, b), Zaracho & Lamas (2008), Hamann et al. (2009), Lamas & Lunaschi (2009), Hamann et al. (2010), Santos & Amato (2010), Hamann et al. (2012), Silva & Müller (2012), Zaracho et al. (2012), Hamann et al. (2013); Smales (2013), Hamann et al. (2014), Graça et al. (2017), Martins-Sobrinho et al. (2017), Quirino et al. (2018), González et al. (2019), Dos Santos Mesquita et al. (2020), Aguiar et al. (2021) in De Oliveira et al. (2022), Cuellar et al. (2022), Silveira et al. (2022), Coimbra et al. (2023), Prata et al. (2023) and present study.

## Discussion

*Centrorhynchus* spp. have been recorded as parasites of *A. catesbeiana* in the Americas only within the native range of distribution the bullfrog (Brandt, 1936; Hollis, 1972). The rates of infection with *Centrorhynchus* cystacanths in *A. catesbeiana* in southern Brazil were higher than those observed by Hollis (1972) (P % = 2.89; MII = 1) in the bullfrog's native range in Texas (USA). However, the prevalence was lower than that recorded by Brandt (1936) (P % = 81.8; MII = 13.90) in the eastern USA. The variations observed in infection rates may be associated with the characteristics of each location, where different factors (biotic and abiotic) may or may not favor transmission and infection rates in *A. catesbeiana*.

Anurans can become infected with *Centrorhynchus* cystacanths through ingestion of parasitized arthropods (intermediate hosts) and by the predation of other parasitized anuran species (paratenic or transport hosts) (Lunaschi & Drago, 2010). In addition, *A. catesbeiana* can exhibit cannibalistic behavior, preying mainly on its juveniles and tadpoles (Barrasso et al., 2009; Leivas et al., 2012; Jancowski & Orchard, 2013; Quiroga et al., 2015), a factor that can contribute to infection by *Centrorhynchus* species.

The higher prevalence of cystacanths in adult anurans may be associated with the predatory potential of larger frogs, and due to the fact that older anurans had longer exposure to infective forms and have larger body area for parasite colonization (Muzzall, 1991; Quiroga et al., 2015). Muzzall (1991) and Andrews et al. (1992) observed in native areas that larger individuals of *A. catesbeiana* showed greater parasite diversity.

Predators of adult specimens of the bullfrog are scarce in the regions where it has been introduced because of its large size (Maneyro & Carreira, 2012). However, in south America, carnivorous mammals such as *Lycalopex gymnocercus* and *Cerdocyon thous* (Canidae), and birds such as *Pitangus sulphuratus* (Tyrannidae), *Rupornis magnirostris* (Accipitridae), *Guira guira* (Cuculidae) and *Athene cunicularia* (Strigidae) are species that which include medium to large-sized anurans as part of their diet (Gatti et al., 2006; Vieira & Teixeira, 2008; Repenning et al., 2009; Rocha et al., 2011; Rodrigues et al., 2011; Soave et al., 2008; Maneyro & Carreira, 2012; Schalk & Morales, 2012; Corrêa et al., 2013) and that have been recorded as definitive hosts of *Centrorhynchus* spp. (Table 3). This suggests that these mammals and birds may prey on adults specimens *A. catesbeiana* and thus contribute to the continuity of *Centrorhynchus* species' life cycle.

*Aquarana catesbeiana* can also participate in the *Centrorhynchus* sp. cycle as a primary transport host, which can be preyed upon by a secondary transport host, and the latter is then consumed by the definitive host. Silva & Ribeiro (2009) reported occurrences of *A. catesbeiana* being preyed upon by *L. latrans*, which has been recorded as a host for *Centrorhynchus* sp. cystacanths (Table 4). *Leptodactylus latrans* is a food resource for birds such as *Rupornis magnirostris* (Accipitridae) and *Tyto furcata* (Strigidae) (Maneyro & Carreira, 2012; Brentano et al., 2020), which are definitive hosts for *Centrorhynchus* species (Table 3). In this context, anurans play an important role in the transmission chain of species within this group of Acanthocephala.

The checklist presented here has expanded the information of previously published lists (Travassos, 1926; Smales, 2007a, b; Ávila & Silva, 2010; Lunaschi & Drago, 2010; Smales, 2013; Campião et al., 2014; Fugassa, 2015; Cuellar et al., 2022), as it has brought together new records for South America, where birds are the definitive hosts most frequently parasitized by *Centrorhynchus* spp. The reports of cysthacanths of *Centrorhynchus* sp. predominated in paratenic hosts in this region, which do not reach the adult stage in amphibians and reptiles, and do not have all their body structures fully formed, which makes identification at the species level difficult (Cabrera-Guzmán & Garrido-Olvera, 2014). The records show that many species of *Centrorhynchus* have yet to be described in South America and, in this regard, use of molecular tools and integrative taxonomic studies are important for the understanding of the diversity of species in this group.

We report for the first time *A. catesbeiana* parasitized by *Centrorhynchus* cystacanths in South America. Bullfrogs can become an important transmission link for *Centrorhynchus* species, considering that many anuran species have been recorded as paratenic hosts (48 spp., see checklist) and are part of the species' diet (Barrasso et al., 2009; Boelter et al., 2012; Maneyro & Carreira, 2012; Leivas et al., 2012; Quiroga et al., 2015; Oda et al., 2019). Considering that bullfrogs they were introduced and also conquered new areas, thereby increasing their geographical distribution in South America (Giovanelli et al., 2008; Laufer et al., 2008; Barrasso et al., 2009; Both et al., 2011; Nori et al., 2011; Iñiguez & Morejón, 2012; Laufer et al., 2021) the records can be expanded through future studies.
